# Insecticide-treated net effectiveness at preventing *Plasmodium falciparum* infection varies by age and season

**DOI:** 10.1186/s12936-017-1686-2

**Published:** 2017-01-17

**Authors:** Andrea G. Buchwald, Jenna E. Coalson, Lauren M. Cohee, Jenny A. Walldorf, Nelson Chimbiya, Andy Bauleni, Kondwani Nkanaunena, Andrew Ngwira, John D. Sorkin, Don P. Mathanga, Terrie E. Taylor, Miriam K. Laufer

**Affiliations:** 1 Institute for Global Health, University of Maryland School of Medicine, 685W, Baltimore St. HSF-1 Room 480, Baltimore, MD 21201 USA; 2 University of Michigan School of Public Health, 1415 Washington Heights, Ann Arbor, MI 48109 USA; 3 University of Malawi College of Medicine, Chichiri, Private Bag 360, Blantyre, Malawi; 4 Department of Osteopathic Medicine, Michigan State University, West Fee Hall, 909 Fee Road, Room B305, East Lansing, MI 48824 USA

**Keywords:** Insecticide-treated nets, *Plasmodium falciparum*, Universal distribution campaign, School-aged children, Malawi

## Abstract

**Background:**

After increasing coverage of malaria interventions, malaria prevalence remains high in Malawi. Previous studies focus on the impact of malaria interventions among children under 5 years old. However, in Malawi, the prevalence of infection is highest in school-aged children (SAC), ages 5 to 15 years. This study examined the interaction between age group and insecticide-treated net (ITN) use for preventing individual and community-level infection in Malawi.

**Methods:**

Six cross-sectional surveys were conducted in the rainy and dry seasons in southern Malawi from 2012 to 2014. Data were collected on household ITN usage and demographics. Blood samples for detection of *Plasmodium falciparum* infection were obtained from all household members present and over 6 months of age. Generalized linear mixed models were used to account for clustering at the household and community level.

**Results:**

There were 17,538 observations from six surveys. The association between ITN use and infection varied by season in SAC, but not in other age groups. The adjusted odds ratio (OR) for infection comparing ITN users to non-users among SAC in the rainy season and dry season was 0.78 (95% CI 0.56, 1.10) and 0.51 (0.35, 0.74), respectively. The effect of ITN use did not differ between children under five and adults. Among all non-SACs the OR for infection was 0.78 (0.64, 0.95) in those who used ITNs compared to those that did not. Community net use did not protect against infection.

**Conclusions:**

Protection against infection with ITN use varies by age group and season. Individual estimates of protection are moderate and a community-level effect was not detected. Additional interventions to decrease malaria prevalence are needed in Malawi.

**Electronic supplementary material:**

The online version of this article (doi:10.1186/s12936-017-1686-2) contains supplementary material, which is available to authorized users.

## Background

Malawi has year-round high transmission of malaria. In 2013 the incidence of clinical malaria was estimated at 4.5 million in a population of 17 million [1, 2]. Insecticide-treated nets (ITNs) have been demonstrated to reduce parasite transmission and prevalence in some sub-Saharan African settings [3–7]. Studies in Malawi have largely found net use to be associated with protection from illness [8–10]. However, infection prevalence has not decreased significantly since scale-up of malaria interventions, including a universal long-lasting ITN distribution campaign in 2012 [11–13]. Malawi lags behind most of its neighbours for decreases in hospital admissions and deaths due to malaria [2]. Understanding the factors contributing to the lack of impact of interventions on infection prevalence is vital to determining appropriate measures to bring about reduction of the malaria burden in Malawi.

School-aged children (SAC), aged 5–15 years, make up ~30% of the population, and recent data from Malawi show that they have the highest infection prevalence, at 4.8 times the odds of infection compared to other ages [14]. Little is known about the effectiveness of ITNs at preventing infection in SAC and how it compares to the effectiveness in other age groups [15]. With the exception of the universal ITN distribution campaign in 2012, most ITN distribution programmes focus on pregnant women and children under 5 years old. Studies on the impact of ITN distribution and use generally study parasite prevalence or disease rates in children under 5 years of age [16–18]. Previous studies have found that even after universal net distribution, SAC were significantly less likely to use ITNs compared to other age groups [19]. Low ITN use among high-prevalence populations such as SAC, even while ITN use increases among other demographics, may contribute to the lack of decline in malaria transmission in Malawi. When previous studies have included wider age ranges, with children both under and over five, the results for impact of community ITN ownership and household ITN ownership on population-wide infection prevalence have been non-significant [20–22]. This may be related to behavioural differences in net use between age groups or persistence of prevalent infections among SAC. Given the contribution of SAC to overall *Plasmodium* prevalence and the age-based differences in behaviour and exposure there is a need for research into age-related variations in the impact of ITN use on infection prevalence.

This analysis used data from six cross-sectional studies conducted over 3 years to evaluate factors that may impact the effectiveness of ITN use for preventing infection. Mixed models were developed that explore the interaction between age and ITN use for prevention of infection, adjusting for season and transmission intensity. An additional model was developed to evaluate the age-specific impact of community ITN use on parasite prevalence in the community. The potential for age-based effect modification of intervention efficacy is important to consider. If there are age differences in the impact of ITN use on infection, this may explain the relative failure of ITN distribution to reduce malaria prevalence in Malawi and have significant impact on the design of future interventions.

## Methods

### Survey

Data come from six cross-sectional household surveys conducted in southern Malawi. Two surveys, the first at the end of the rainy, high transmission season (April–May), and the second at the end of the dry, low transmission season (September–October), were conducted each year from 2012 to 2014 [14]. Three-hundred households were selected from each of three districts using two-stage cluster sampling [23]. Districts included in the study were Blantyre (an urban, low transmission setting), Chikhwawa (a rural, low altitude, high transmission setting), and Thyolo (a rural, high altitude, low transmission setting). Using probability proportionate to size, 10 enumeration areas (EA) were randomly selected within each district. Households were selected from each EA by compact segment sampling [23]. Each selected EA was divided into segments each containing approximately 30 households. One segment from each EA was randomly selected and all households within that segment were visited (referred to as a community in the analysis). The same selected communities in each district were surveyed each season, and all households within a given community were visited on a single day. Households were excluded if there were no adults over 18 years old present to provide consent. If excluded, a household was replaced with the nearest household within the compact segment, this household was selected by convenience. A universal net distribution campaign occurred in Malawi in 2012 after the first survey. The goal of the distribution campaign was to distribute nets to all households at a ratio of one net for every two people in the household.

### Ethical treatment of human subjects

Prior to study initiation, permission to survey each village was provided by the village leaders. Written informed consent was obtained for all adults and children and assent was obtained for children age 13–17 years. All questionnaires were administered in the local language. The study received ethical approval from both the University of Maryland Baltimore and Michigan State University Institutional Review Boards and the University of Malawi College of Medicine Research and Ethics Committee.

### Study participants

Members of a household were defined as individuals who slept in the house for at least two weeks of the previous month. Data about all household members were collected from adult household members; blood specimens were collected from household members over 6 months of age who were present at the time of survey. Data was not collected on individuals who did not qualify as members of a household. Households in the same geographic area were selected in each survey, and the same households may have been visited repeatedly, however, it was not possible to collect identifying data to track individuals or households between surveys. The specific individuals who answered survey questions varied from survey to survey depending on those present on the day of the survey.

### Data collection and variable definitions

Survey questionnaires were adapted from the standardized Malaria Indicator Survey tools [11]. Data were collected on android-based tablets using OpenDataKit and managed using Research Electronic Data Capture (REDCap) tools [24]. Data collected included household characteristics and socio-economic indicators, individual demographics, household net ownership and individual net use. Participants were initially classified into three age groups, defined a priori, for preliminary analysis: children under five, SAC (children 5–15 years old), and adults (over 15 years). SAC were later further divided into two categories for final analysis: young SAC (ages 5–9) and older SAC (ages 10–15).

The primary outcomes of interest were the odds of individual *Plasmodium falciparum* infection and community infection prevalence. Real-time PCR (rtPCR) performed on dried blood spots collected on 3M Whatman filter paper and microscopic examination of blood smears collected at the time of survey were used to determine presence or absence of *Plasmodium* infection. Individuals were considered parasite positive by rtPCR if blood samples were positive for the *P. falciparum* lactate dehydrogenase gene [25]. A secondary analysis used blood smear results to determine presence of microscopically detectable infection. Thick blood smears were stained with Field’s or Giemsa stain and read by at least two independent readers. Individuals were considered parasite positive by microscopy only if presence was confirmed by at least two readers. Community infection prevalence was defined as the per cent of specimens positive for *P. falciparum* in a given community at a given survey. Site transmission intensity (‘transmission setting’) was defined using the average community prevalence by rtPCR from all six surveys. Prevalence among children under five was tested as an alternate method for defining transmission setting but the variable was zero-inflated. Tertiles of low, moderate and high transmission communities were defined as having less than 7% prevalence, from 7 to 11% prevalence and over 11% prevalence, respectively.

The primary exposures of interest were individual, household and community net use. Survey staff asked to observe all bed nets in the household at the time of survey. An individual was categorized as using a particular bed net if they were identified in the question “Which members of the household slept under this bed net last night?” Household net use was defined as the proportion of household members reported to have slept under a net the night before the survey and was categorized as high (80% or greater), low (<80%) or none based on the Malawi Malaria Strategic Plan recommendations [26]. Household net use was alternately examined as a linear variable, but did not change the results. The categorical variable was chosen for final models as it is more easily interpretable for public health purposes. Community prevalence of net use was defined as the proportion of individuals in the community reported to have slept under a net the night before the survey and was categorized as high (80% or higher), or low (<80% net use).

A household wealth index, broken down into tertiles, was developed using principal components analysis, including ownership of common economic indicators, presence of electricity in the household, income category, and food security; wealth index was calculated for each of the households in the study population. Head of household education level was classified into two categories: having some secondary education or more, and no secondary education.

### Statistical analysis

All statistical analyses were run twice, defining the outcome of interest alternately as *P. falciparum* infection detected (1) by rtPCR and, (2) microscopically. Two sets of analyses were run. The first examined the association between net use and individual infection, and the second examined the association between community net use and prevalence of infection.

### Association between individual net use and individual infection

Multivariable mixed effect logistic regression (SAS PROC GLIMMIX) was used to model the association between infection with malaria parasites and individual ITN use (predictor variable of interest), adjusted for potential confounders. The analyses accounted for clustering at the household and community level using nested random intercepts. Potential confounders were tested for inclusion in the model if in bivariate analysis they were associated with individual infection and were known from previous work [19] to be associated with individual ITN use (transmission setting, age category, ratio of nets to sleeping spaces, proportion of nets hanging in a household, head of household education level, household wealth index, and person to net ratio). The following additional variables were included in the multivariable analyses due to association with both infection and ITN use: season (rainy vs dry season), household net use and community net use. A variable for community infection prevalence at the time of each survey was included in multivariable statistical models to adjust for local prevalence differences within transmission settings and between seasons. All final models included individual net use, community net use, household net use, wealth index, age group, and community infection prevalence. Interaction terms were included to test for modification of the association between net use and infection by age, by presence of a school-age child in the household, and by season. If presence of interaction was suspected, stratified models were constructed. Final analysis was done in the following three strata: SAC in the rainy season, SAC in the dry season, and non-SAC. In analyses among non-SAC, SAC were excluded from the pool of study subjects and variables for the presence of a SAC in the household, and SAC ITN use were included.

### Association between community net use and prevalence of infection

Linear mixed models (PROC MIXED) were constructed to examine the crude association between community ITN use and community prevalence of infection. These models were used to assess the bivariate association between all independent variables and community infection prevalence, as well as the bivariate association between covariates and community ITN use. One-way ANOVAs were used to compare infection prevalence within levels of categorical variables. Variables were retained in the final model if they had *p* value <0.05 after all other variables were added, or if addition of the variable qualitatively changed the estimate for ITN use and if there was not excessive collinearity with any other variable in the model (identified when high variance inflation was present). Community prevalence of infection has a right skewed distribution so community parasite prevalence was log transformed. Various covariance structures were tested to account for the serial autocorrelation of repeated measures obtained from the same community. Criteria for selection of covariance structure were parsimony (models with fewer covariance parameters were preferred) and decreasing Akaike’s information criteria. Variables chosen a priori to test for inclusion in this analysis based off causal assumptions were community prevalence of net use, proportion of children under five who used nets, proportion of SAC who used nets, average community wealth index, proportion of community population comprised of children under five (to account for population structure), and transmission setting. Mixed models included random effects for variation in baseline community prevalence (random intercept) and for community-level variation in slope over time to account for repeated measurement of community level variables over time. All a priori selected variables were tested for inclusion in the final model. All adjusted models included community net use, season and transmission setting.

All analyses were conducted with SAS 9.4.

## Results

There were 22,132 individuals from six surveys with ITN use data collected (Table 1). Population characteristics did not vary significantly between surveys; average household size was 4.1 ± 1.7 (mean ± SD); 55% of the surveyed population was female; in approximately 25% of households, the head of household had attended some secondary school or more, and in 60% of households, the head of household had not completed primary school. Community structure remained constant over time, with approximately 30% of all respondents in the SAC age group at each time point. Net ownership, net use, and parasite prevalence differed (Chi square p < 0.001) across surveys.

**Table 1:** Population characteristics from six cross-sectional surveys (N = 22,132)

Population characteristics	Rainy 2012	Dry 2012	Rainy 2013	Dry 2013	Rainy 2014	Dry 2014
N	3475	3873	3767	3680	3590	3747
Low transmission	900	1049	968	973	943	1011
Moderate transmission	1425	1563	1561	1485	1468	1520
High transmission	1150	1261	1238	1222	1179	1216
Age category						
Under 5 years (n = 3856)	17.7%	17.7%	17.8%	16.8%	17.5%	17.0%
5–15 years (n = 7358)	30.2%	31.7%	33.3%	34.4%	33.9%	36.0%
Over 15 years (n = 10,918)	52.1%	50.6%	48.9%	48.8%	48.6%	47.0%
Per cent of households that own one or more nets^b^	51.4%	83.1%	87.5%	83.2%	81.3%	70.9%
Household net ownership						
Low transmission^b^	55.7%	75.7%	82.6%	79.8%	75.4%	72.6%
Moderate transmission^b^	51.8%	80.1%	86.0%	84.7%	84.9%	69.5%
High transmission^b^	47.4%	92.7%	93.4%	84.3%	81.7%	71.2%
Per cent of children under 5 who use nets^b^	47.0%	68.1%	77.8%	66.6%	77.7%	53.1%
Per cent of school-aged children who use nets^b^	24.1%	49.2%	59.4%	45.5%	52.0%	33.0%
Per cent of adults over age 15 who nets^b^	37.1%	60.6%	73.7%	60.9%	68.5%	49.4%
Parasite prevalence (rtPCR)						
Children under 5^b^	10.8%	7.7%	13.0%	5.6%	17.0%	9.8%
School-aged children^b^	28.3%^a^	21.9%^a^	28.4%^a^	17.5%^a^	24.2%^a^	18.2%^a^
Adults over age 15^b^	13.4%	8.1%	13.5%	7.4%	17.2%	9.0%
Parasite prevalence (microscopy)						
Children under 5^b^	8.9%	5.7%	10.3%	5.1%	10.0%	8.1%
School-aged children^b^	19.7%	10.5%	22.1%	11.1%	17.8%	10.8%
Adults over age 15^b^	6.6%	2.7%	7.3%	3.2%	8.8%	3.1%

^a^Indicates SAC have significantly higher parasite prevalence than other age categories

^b^Indicates variables significantly different at an alpha level of 0.001 across surveys

**Fig. 1:**
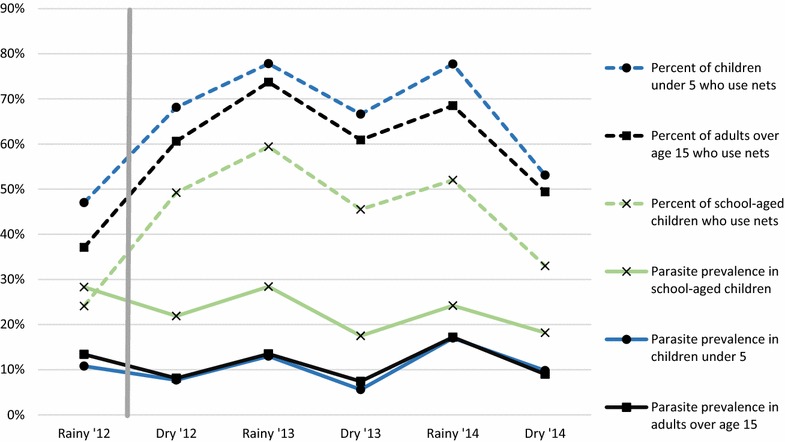
Net use and infection prevalence (PCR) by age and by survey. *Vertical line* indicates the timing of universal net distribution in Malawi

Among all surveys, 17,538 individuals (79.2%) had dried blood spots available for rtPCR and 17,586 (79.5%) had microscopy results. Individuals over the age of 15 were less likely to have been present at the time of survey to contribute blood samples than other age groups (p < 0.01). Only individuals contributing blood samples were included in further analyses. The total prevalence of infection from rtPCR and microscopy across all six surveys was 15.2 and 9.2%, respectively. SAC were more likely to have infections than all other age groups at all time-points (Table 1, Fig. 1); 47.3% of all PCR-identified infections were undetected by microscopy with 33.5, 37.9 and 57.0% of infections being sub-microscopic among children under age five, SAC, and adults, (p < 0.0001), respectively.

ITN ownership increased significantly (p < 0.0001) after universal net distribution in 2012, from 51% in the first survey to 83% in the second survey. ITN use also increased significantly after universal net distribution between the first and the third surveys in all age categories.

### Association between net use and PCR-detected infection in individuals

In bivariate analysis (Additional file 1: Table S1), covariates which were associated with infection were net use, season, age category, gender, head of household education level, presence of a child under the age of five in the household, presence of a SAC in the household, household wealth index, household net use, proportion of nets to people in a house, proportion of EA using nets, and transmission setting.

The crude odds ratio (OR) for infection comparing individuals who used nets to those who did not was 0.75 (95% CI 0.68, 0.83). There was evidence of interaction with ITN use by season and by age category in adjusted models (p for interaction = 0.04 and 0.02, respectively). The association between net use and infection did not differ for children under five and adults over 15 years of age (p for interaction = 0.81) so they were analysed together and defined as non-SAC (Table 2). In age-category stratified models, there was evidence of seasonal effect modification only among SAC (p for interaction = 0.03). All subsequent analysis was done in the following three strata: SAC in the rainy season, SAC in the dry season, and non-SAC.

**Table 2:** Adjusted odds ratios for association between covariates and infection (qPCR) using mixed models

Adjusted models	SAC rainy seasons	SAC dry seasons	Non SAC
Individual net use	0.78 (0.56, 1.10)	0.51 (0.35, 0.74)	0.78 (0.64, 0.95)
No net use	Ref	Ref	Ref
Household net use > 80%	1.24 (0.88, 1.76)	1.18 (0.80, 1.75)	1.12 (0.93, 1.36)
Net use <80%	Ref	Ref	Ref
Community net use > 80%	1.03 (0.80, 1.34)	1.39 (1.01, 1.91)	1.13 (0.95, 1.34)
Net use <80%	Ref	Ref	Ref
Wealth index (one unit increase)	0.90 (0.86, 0.94)	0.90 (0.85, 0.95)	0.92 (0.87, 0.95)
Male gender	1.32 (1.08, 1.62)	Not included	Not included
Female	Ref	–	–
Under age 5	–	–	1.10 (0.96, 1.27)
Age 15 and older	–	–	Ref
SAC in household	–	–	0.80 (0.69, 0.92)
No SAC in household	–	–	Ref
SAC age 5–9	0.70 (0.57, 0.86)	0.67 (0.54, 0.83)	–
SAC age 10–15	Ref	Ref	–

All models adjusted for ea prevalence at the time of survey and survey number

**Fig. 2:**
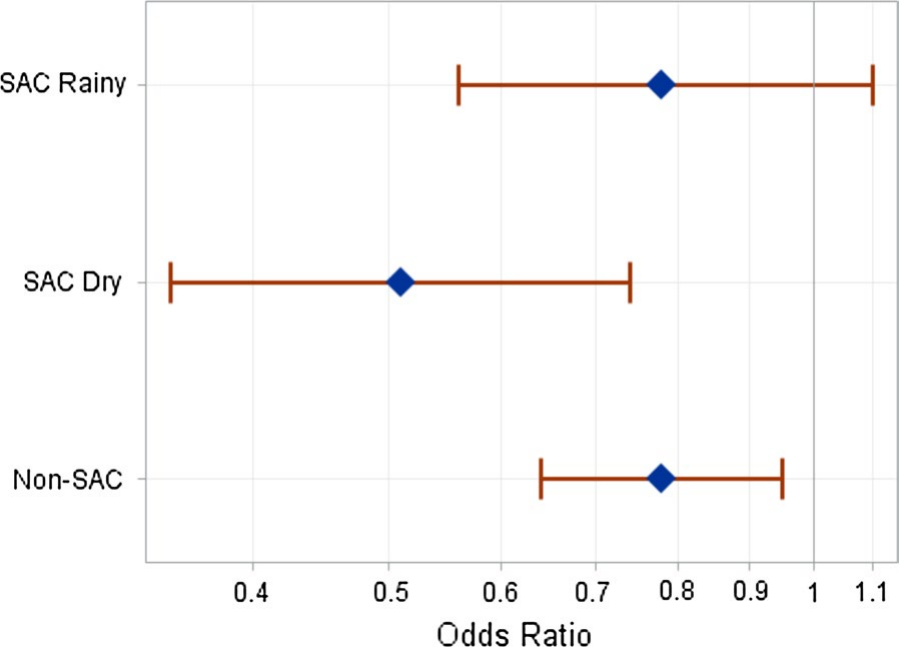
Adjusted odds ratios for infection comparing ITN users to non-users by strata. *Diamonds* indicate strata-specific odds ratio estimate*. Red horizontal bars* indicate 95% confidence intervals. *Grey vertical line* at 1 indicates an odds ratio of 1, or no difference between ITN users and non-users

In adjusted models, among SAC in the rainy season, the OR of infection for net users compared to those who did not use nets was 0.78 (95% CI 0.56, 1.10) (Table 2; Fig. 2). SAC net use in the dry season was associated with a 49% decrease in the odds of infection (OR = 0.51 (95% CI 0.35, 0.74), p < 0.001). Among non-SAC, net use was associated with a 22% decrease in odds of infection in all seasons (OR = 0.78 (95% CI 0.64, 0.95), p = 0.01). Variables associated with increased odds of infection in at least one of the stratified adjusted models were high community net use, decreasing household wealth index, male gender, decreasing education level of household head, lack of SAC in the household, and older SAC age category (aged 10–15) (Table 2). Neither per cent of household using nets nor dichotomous household net ownership were strongly associated with decreased odds of infection in any stratified models.

### Association between community net use and prevalence of PCR-detected infection

There were 30 communities sampled over six time points. Comparing results from the rainy season surveys from before (2012) and after (2013) the universal net distribution campaign, despite increases in net use (Chi square p value <0.0001), there was no decrease in infection prevalence (Table 1). In crude analysis (Table 3), a 10% increase in community net use was associated with a 6% (SE = 2%, p = 0.02) increase in infection prevalence. Other variables strongly associated with increasing prevalence in preliminary analysis were increasing proportion of SAC net use, decreasing wealth index and higher transmission setting. After adjustment for season, transmission setting, community wealth index, and community net use, only community net use remained strongly associated with infection prevalence (Table 3).

**Table 3:** Crude and adjusted estimates of percent change in infection prevalence^a^ associated with net use and covariates

Covariate	Unadjusted estimate		Adjusted estimate^a^	
	% Change (SE)	p value	% Change (SE)	p value
Community net use (10% increase)	6 (2)	0.02	6 (2)	0.01
Proportion of children under 5 using nets (10% increase)	3 (2)	0.12	−4 (4)	0.36
Proportion of population composed of children under 5 (10% increase)	−8 (16)	0.64	−1 (5)	0.97
Proportion of SAC who use nets (10% increase)	5 (2)	0.01	5 (5)	0.33
Average community wealth index (1 unit increase)	−18 (7)	0.01	−7 (4)	0.09
Transmission setting				
Low transmission	Ref	–	Ref	–
Moderate transmission	85 (16)	<0.001	76 (16)	<0.001
High transmission	209 (17)	<0.001	192 (19)	<0.001

All models include random effect for EA and account for correlation across surveys using a Toeplitz covariance structure

Adjusted models all include community net use, average community wealth index, season, and transmission setting

^a^Infection prevalence measured by molecular methods and log transformed for analysis

### Individual and community-level association with net use and microscopically detected infection

Results from all analyses using microscopically detected infections were similar to results using rtPCR-detected infections (Additional file 1: Tables S2, S3). However, results using microscopy varied from rtPCR results in that ITN use was more closely associated with protection (significant at a p < 0.05 level) against individual level infection in all three strata in adjusted models (Additional file 1: Table S2).

## Discussion

This study found evidence of effect measure modification in the relationship between ITN use and *P. falciparum* infection by both age and by season. Using repeated cross-sections of randomly selected communities, nets were highly protective among SAC during the dry season, yet provided weaker protection from infection during the rainy seasons in this age group. This relationship in SAC differed from that seen in children under five and adults over 15, among whom ITN use was associated with a statistically significant 22% decrease in infection year round, confirming previous findings among children under five in Malawi [8]. Previously published results from these cross-sectional surveys [19] have found that SAC consistently used nets less frequently than children under five and adults, even after the universal distribution campaign. As patterns of net use between age groups remain the same over time and by season, other hypotheses must be explorde to explain the results. The underlying mechanisms driving this effect modification are unknown but may be related to multiple factors including mosquito density, behaviours prior to net use, acquired immunity, and the high prevalence of prolonged asymptomatic infections among SAC [14, 27].

### Transmission intensity

Mosquito density is notably lower during the dry season than the rainy season, and ITN use at night in the dry season may protect against a larger relative proportion of mosquitoes than during the rainy season. Transmission intensity has previously been demonstrated to modify intervention efficacy [4]. The seasonal difference in ITN effectiveness among SAC could be related to wide variation in mosquito density (and thus transmission intensity) between the rainy and dry seasons. With increasing insecticide resistance in Malawi [28], ITNs may only be able to protect against a small proportion of mosquitoes. As mosquito density increases, ITNs may fail more frequently. Additionally, vectors may have more restricted biting behaviours in the dry season and individuals may be less likely to be bitten outside of sleeping hours.

### Age, behaviour and acquired immunity

The differing effectiveness of ITNs by age group may have to do with a combination of behavioural differences and acquired immunity. Behavioural differences may play a role in the age-based effect modification this study found. In the rainy season, when a greater proportion of individuals overall use ITNs [19], SAC may be more likely to use lower quality, older ITNs which are less effective [29]. Additionally, due to a development of immunity to disease but not infection, a high proportion of infections detected in SAC may be persistent, asymptomatic infections. There is evidence that SAC may carry infections for a longer duration than other ages [27]. A high prevalence of persistent asymptomatic infection could contribute to the apparent lack of effectiveness when measuring net use using cross-sectional surveys. If SAC carry a high prevalence of persistent infections, ITN use would appear less effective in comparison to a population where a larger proportion of infections detected are incident.

Due to the high risk of malaria mortality at young ages, the majority of previous studies on efficacy of ITNs focus on children under five [7–10, 13]. Findings from this study are in agreement with one previous study from Malawi, in which no statistically significant protective effect of ITNs was detected when including children of all ages. However, the study did not address effect modification related to age [22]. Studies in other settings that have included SAC found either varying efficacy of ITNs among SAC [30, 31] or varying efficacy by age [32]. By evaluating effect modification by age, this study was able to identify heterogeneity in the efficaciousness of ITNs by age group and season, perhaps explaining some of the inconsistency in magnitude of previous results.

### Microscopy versus molecular detection of infection

Previous studies examining the effect of ITN use on infection prevalence have relied on microscopy to measure *P. falciparum* prevalence [10, 13, 30–33], however 47% of all infections detected in these surveys were sub-microscopic and SAC were more likely to have sub-microscopic infections than younger children [14]. Unlike microscopy, rtPCR can detect prevalent low-density asymptomatic infection. In this study ITNs protected against microscopically detectable infections but had weaker associations with PCR-detected infections. Individuals with sub-microscopic infections may still transmit parasites to mosquitoes [34, 35] suggesting microscopy may not be sufficiently sensitive to detect the full effect of ITN use on transmission.

Unique aspects of this study design may have biased the results. Despite having multiple time points, individual-level data are cross-sectional and, although study staff returned to the same location for each survey, analysis was unable to account for the possibility of surveying different individuals over time. Additionally, these data were collected as part of a surveillance programme without control arms and with only one season of pre-intervention data.

A likely explanation for a lack of community effect in this data is residual confounding due to unmeasured environmental variables and mosquito density fluctuation. This study did not collect data on environmental and climatic factors influencing mosquito density and parasite prevalence. Lack of change in community prevalence over time may be confounded by unmeasured environmental factors. Increasing mosquito density leads to increased net use independent of malaria prevalence. In individual-level models, community ITN use may have functioned as a proxy for mosquito density and allowed statistical models to detect a protective effect of individual ITN use on malaria infection prevalence. However, for community-level models, the data did not have an appropriate measure of mosquito density to detect the true protective efficacy of community net use. Increasing community net use was consistently associated with an increase in infection prevalence, however this association is likely not causal as increasing mosquito densities lead both to increasing net use and increasing prevalence. A long-term decline in *P. falciparum* prevalence in Malawi may provide an additional explanation for why it was not possible to find a community-level impact of ITN distribution. Looking at children under five alone, baseline prevalence in this study was significantly less than that seen in previous studies in Malawi [36, 37].

Individuals excluded from the study for not meeting the definition of “member of household” were most likely to be adult males. There was no data collected data on net use for individuals sleeping away from home, only data on those that were sleeping regularly in the household at the time of survey. While this could potentially effect the generalizability of the study results as these results could not be applied to individuals who move around frequently and have no stable household, this would be unlikely to influence the results among the population studied.

## Conclusion

In this study, ITN use did not have a uniform impact in all ages, with seasonal variation among SAC. This study found an association between ITN use and infection, however the association was modest. Among SAC, a population carrying the majority of *P. falciparum* infections in Malawi, the association between ITN use and infection was diminished during the rainy season using molecular detection methods. This study adds evidence to a growing body of literature suggesting that SAC may represent an important reservoir population. Unique qualities of both intervention use and the nature of infection in SAC may allow them to serve as a reservoir of infection in malaria-endemic regions. Future studies should include data from SAC and consider the possibility of effect modification by age when examining intervention effectiveness.

Despite the possibility of residual confounding due to changing mosquito densities and other ecological factors, there was no decrease in malaria prevalence after universal net distribution and no decrease in malaria associated with increasing community net use. Current surveillance methods using single time-point cross-sectional surveillance of children under five using microscopy alone may fail to capture the impact of ITN use on malaria prevalence in a community. The findings from this study imply that ITN distribution alone may not be sufficient to decrease *P. falciparum* transmission in Malawi and other methods of prevention are required.

## Additional file

**Additional file 1: Table S1.** Predictors of infection (qPCR positive) stratified by net use (n=17,538). **Table S2.** Adjusted odds ratios for association between covariates and infection (microscopy positive) using mixed models. **Table S3.** Crude and adjusted estimates of ln (microscopy positive prevalence) difference associated with covariates.

## Abbreviations

EA: enumeration area
ITN: insecticide-treated net
OR: odds ratio
CI: confidence interval
SAC: school-aged children
rtPCR: real time polymerase chain reaction
SD: standard deviation
