# Supplementary material for: Insecticide-treated net effectiveness at preventing Plasmodium falciparum infection varies by age and season
Source: Malar J. 2017 Jan 17;16:32. doi: 10.1186/s12936-017-1686-2 (PMC5240228; doi:10.1186/s12936-017-1686-2)
Supplement: Supplementary file 1 — Additional file 1: Table S1. Predictors of infection (qPCR positive) stratified by net use (n=17,538). Table S2. Adjusted odds ratios for association between covariates and infection (microscopy positive) using mixed models. Table S3. Crude and adjusted estimates of ln (microscopy positive prevalence) difference associated with covariates. [file 12936_2017_1686_MOESM1_ESM.docx]

**SUPPLEMENTARY TABLES**

Table S1. Predictors of infection (qPCR positive) stratified by net use (n=17,538)

| **Characteristic** | | **Percent of Individuals Positive for Parasites** | | |
| --- | --- | --- | --- | --- |
|  |  | **Total** | **No Net Used** | **Net Used** |
| **Net use the night before survey**** | |  |  |  |
|  | **Did not use a net (n=7772)** | 16.7% | 16.7% | - |
|  | **Used a net (n=9766)** | 14.0% | - | 14.0% |
| **Age**** | |  |  |  |
|  | **Under 5 (n=3335)** | 10.7% | 10.9% | 10.6% |
|  | **5 – 15 (n=6159)** | 22.7% | 24.2% | 20.9% ^a^ |
|  | **Over 15 (n=8044)** | 11.4% | 11.2% | 11.5% |
| **Sex**** | |  |  |  |
|  | **Female (n=10701)** | 14.0% | 15.1% | 13.2% |
|  | **Male (n=6803)** | 17.2% | 19.2% | 15.5% |
| **Head of household education level**** | |  |  |  |
|  | **Never attended (n=4063)** | 21.7% | 22.7% | 20.6% |
|  | **Some Primary (n=7095)** | 17.1% | 17.5% | 16.8% |
|  | **Completed Primary (n=2387)** | 11.2% | 13.1% | 10.0% |
|  | **Some Secondary or more (n=3993)** | 7.7% | 9.0% | 7.0% |
| **Child under age 5 in the household**** | |  |  |  |
| **No (n=6138)** | | 14.0% | 16.1% | 11.9%^a^ |
| **Yes (n=8065)** | | 18.0% | 19.2% | 17.1% |
| **School-aged child in the household*** | |  |  |  |
|  | **No (n=3590)** | 12.4% | 11.9% | 12.6% |
|  | **Yes (n=7789)** | 10.6% | 10.8% | 10.5% |
| **Household wealth index**** | |  |  |  |
|  | **Lowest (n=4692)** | 20.1% | 20.2% | 20.1% |
|  | **Middle (n=8176)** | 16.8% | 18.0% | 16.0% |
|  | **Highest (n=4670)** | 7.5% | 8.5% | 6.9% |
| **Household member net use*** | |  |  |  |
|  | **Over 80% net use in household (n=7197)** | 15.2% | 24.0% | 15.0% |
|  | **Between 0 and 80% (n=4964)** | 14.6% | 18.5% | 11.4% |
|  | **0% net use in household (n=5377)** | 15.8% | 15.8% | - |
| **Ratio of people in house to nets**** | |  |  |  |
|  | **No nets in house (n=3660)** | 16.2% | 16.2% | - |
|  | **More than 3 people per net (n=5179)** | 16.3% | 18.6% | 14.3% |
|  | **3 people per net (n=2646)** | 16.9% | 18.4% | 16.5% |
|  | **Less than 3 people per net (n=6053)** | 13.0% | 13.2% | 12.9% |
| **Portion of EA using nets**** | |  |  |  |
|  | **Lowest < 40% (n=4331)** | 13.0% | 14.7% | 9.1% |
|  | **Moderate 40% - 80% (n=8426)** | 14.0% | 16.8% | 11.9% |
|  | **Highest < 80%(n=4781)** | 19.4% | 22.2% | 18.5% |
| **Transmission Setting**** | |  |  |  |
|  | **Low Transmission (n=4515)** | 4.1% | 4.8% | 3.6% |
|  | **Moderate Transmission (n=6980)** | 8.8% | 9.9% | 8.0% |
|  | **High Transmission (n=6043)** | 30.9% | 33.8% | 28.7% |
| **Season** | |  |  |  |
|  | **Rainy Season** | 18.7% | 19.5%^b^ | 18.1% |
|  | **Dry Season** | 12.0% | 14.5% | 9.9% |

** Indicates variable with Chi-square test p-value less than 0.0001

* Indicates variable with Chi-square test p-value less than 0.01

^a^ Indicates variable with Breslow-Day test p-value for interaction less than 0.05

^b^ Indicates variable with Breslow-Day test p-value for interaction less than 0.0001

Table S2. Adjusted odds ratios for association between covariates and infection (microscopy positive) using mixed models

| **Adjusted Models** | **SAC rainy seasons** | **SAC dry seasons** | **Non SAC** |
| --- | --- | --- | --- |
| **Individual net use** | 0.67 (0.-46, 0.97) | 0.57 (0.37, 0.88) | 0.73 (0.56, 0.94) |
| **No net use** | Ref | Ref | Ref |
| **Household net use > 80%** | 1.56 (1.06, 2.30) | 1.13 (0.71, 1.80) | 1.18 (0.92, 1.53) |
| **Net use < 80%** | Ref | Ref | Ref |
| **Community net use >80%** | 1.35 (1.05, 1.74) | 1.07 (0.73, 1.55) | 1.58 (1.31, 1.92) |
| **Ne use < 80%** | Ref | Ref | Ref |
| **Wealth index (one unit increase)** | 0.89 (0.84, 0.94) | 0.90 (0.84, 0.96) | 0.93 (0.89, 0.97) |
| **Male sex** | Not significant | Not included | Not included |
| **Female** | - | - | - |
| **Under age five** | - | - | 1.51 (1.36, 1.92) |
| **Age 15 and older** | - | - | Ref |
| **SAC in household** | - | - | 0.85 (0.71, 1.02) |
| **No SAC in household** | - | - | Ref |
| **SAC age 5 – 9** | Not significant | Not significant |  |
| **SAC age 10-15** | - | - |  |

* All models adjusted for ea prevalence at the time of survey and survey number

Table S3. Crude and adjusted estimates of ln (microscopy positive prevalence) difference associated with covariates

|  | **Unadjusted estimate** | | **Adjusted estimate*** | |
| --- | --- | --- | --- | --- |
| **Covariate** | **Change (SE)** | **p-value** | **Change (SE)** | **p-value** |
| **Community net use (10% increase)** | 0.07 (0.03) | 0.02 | 0.06 (0.03) | 0.04 |
| **Proportion of children under 5 using nets (10% increase)** | 0.07 (0.03) | 0.02 | 0.02 (0.03) | 0.46 |
| **Proportion of population composed of children under 5**  **(10% increase)** | 0.03 (0.20) | 0.86 | 0.02 (0.18) | 0.93 |
| **Proportion of SAC who use nets**  **(10% increase)** | 0.05 (0.03) | 0.08 | -0.04 (0.06) | 0.51 |
| **Average community wealth index**  **(1 unit increase)** | -0.14 (0.08) | 0.07 | 0.01 (0.06) | 0.86 |
| **Transmission setting** |  |  |  |  |
| **Low transmission** | Ref | - | Ref | - |
| **Moderate transmission** | 0.54 (0.23) | 0.03 | 0.51 (0.22) | 0.03 |
| **High transmission** | 1.88 (0.23) | <0.001 | 1.83 (0.23) | <0.001 |

All models include random effect for EA and account for correlation across surveys using a Toeplitz covariance structure

Adjusted models all include community net use, season, and transmission setting.
